# Deciphering the Correlation between Breast Tumor Samples and Cell Lines by Integrating Copy Number Changes and Gene Expression Profiles

**DOI:** 10.1155/2015/901303

**Published:** 2015-07-26

**Authors:** Yi Sun, Qi Liu

**Affiliations:** Department of Central Laboratory, Shanghai Tenth People's Hospital, School of Life Sciences and Technology, Tongji University, Shanghai 200092, China

## Abstract

Breast cancer is one of the most common cancers with high incident rate and high mortality rate worldwide. Although different breast cancer cell lines were widely used in laboratory investigations, accumulated evidences have indicated that genomic differences exist between cancer cell lines and tissue samples in the past decades. The abundant molecular profiles of cancer cell lines and tumor samples deposited in the Cancer Cell Line Encyclopedia and The Cancer Genome Atlas now allow a systematical comparison of the breast cancer cell lines with breast tumors. We depicted the genomic characteristics of breast primary tumors based on the copy number variation and gene expression profiles and the breast cancer cell lines were compared to different subgroups of breast tumors. We identified that some of the breast cancer cell lines show high correlation with the tumor group that agrees with previous knowledge, while a big part of them do not, including the most used MCF7, MDA-MB-231, and T-47D. We presented a computational framework to identify cell lines that mostly resemble a certain tumor group for the breast tumor study. Our investigation presents a useful guide to bridge the gap between cell lines and tumors and helps to select the most suitable cell line models for personalized cancer studies.

## 1. Introduction

Breast cancer is one of the most frequently diagnosed life-threatening cancers in women with about 235,000 new cases expected in the United States in 2014. Breast cancer is a complex and heterogeneous disease such that they may have different prognoses. It responds to therapy differently despite similarities in histological types, grade, and stage. In the laboratory, the breast cancer is often modelled using established breast cancer cell lines due to their ease of being acquired and used [1].

However, accumulated evidences have pointed out the genomic differences between cancer cell lines and tissue samples in the past decades [2–4]. In the review of Holliday and Speirs [1], they demonstrated that cell lines are prone to genotypic and phenotypic drift during their continual culture. This is particularly common in the more frequently used cell lines, especially those that have been deposited in cell banks for many years [5]. Subpopulations may arise and cause phenotypic changes over time by the selection of specific, more rapidly growing clones within a population. Considering these findings, it is essential for researchers to choose the decent cell lines models when designing experiments and interpreting results, especially if such cell lines are regarded as valid models in evaluating the pathobiology of breast cancer and/or the likely response to novel drug therapies [1].

With the quick development of the whole genome sequencing and other “-omics” techniques, now it becomes possible to systematically explore the relationship between tumor tissues and cancer cell lines and identify the cell lines that most closely resemble particular tumor subtypes. In The Cancer Genome Atlas (TCGA), the genome and expression profiles of at least 500 tissue samples per tumor type are being comprehensively characterized [6]. The Broad-Novartis Cancer Cell Line Encyclopedia (CCLE) contains a compilation of gene expression, chromosomal copy number, and massively parallel sequencing data from 947 human cancer cell lines that are used as models for various tumor types [7]. These huge data accumulated regarding tumor samples and cell lines have provided a great potential to mine their associations and characterize the cancer mechanisms.

Traditionally, breast cancer was diagnosed into luminal A, luminal B, HER2+/ER−, basal-like, and normal-like subtypes based on gene expression profiling or immunohistochemical (IHC) characteristic [6]. However, classification criteria defined by using only this information may be not sufficient and likely overly general. In this study, we focus on the primary tumors of breast and try to depict the genomic characteristics of these tumors based on their gene expression profiles. Besides, previous studies have suggested that DNA copy number variations (CNVs) are important influential factors for altered gene expression levels in cancer [8–10]. In a lung cancer study, approximately 78% genes showed a positive correlation between CNV and gene expression level [11]. Considering the potential key constitution of CNVs associated with the gene expression variations in breast tumors, copy number profiles were also incorporated in this study.

Using the genomic information, the relationship between these primary breast tumors and the breast cancer cell lines was explored. Furthermore, as intrinsic differences exist among the breast tumor, we also attempt to figure out the correlation between the cell lines and different breast tumor groups and design an efficient computational framework which helps to select the most suitable cell line models for a specified tumor type.

## 2. Materials and Methods

### 2.1. Data Collection and Tumor Sample Classification

In our study, we only reserved breast tumor samples or cancer cell lines with both genome-wide DNA copy number information and mRNA expression profiles available. As a curation result, 543 primary breast tumor samples (including 52 normal samples) profiled by TCGA [6] and 59 breast cancer cell lines from the CCLE [7] were obtained.

Generally, breast cancer may be categorized into luminal A, luminal B, HER2+/ER−, basal-like, and normal-like subtypes based on gene expression profiling or immunohistochemical (IHC) characteristics [12, 13]. However, large-scale genomics projects have revealed heterogeneities exist within the same class of breast cancer patients defined by the classic grouping [6]. Here, in order to make a relatively consistent molecular background for the tumor samples in the same group, we subdivided the 491 breast tumors into 8 groups according to the presence or absence of expression of the estrogen receptor (ER), the human epidermal growth factor receptor 2 (ERBB2/HER2), and progesterone receptor (PR) in combination, and there are ER group (ER+, PR−, and HER−; *n* = 46), PR group (ER−, PR+, and HER−; *n* = 5), HER group (ER−, PR−, and HER+; *n* = 19), ERPR group (ER+, PR+, and HER−; *n* = 282), ERHER group (ER+, PR−, and HER+; *n* = 14), PRHER group (ER−, PR+, and HER+; *n* = 1), TP group (ER+, PR+, and HER+; *n* = 38), and TN group (ER+, PR+, and HER+; *n* = 86). The PRHER group was removed in the further study as there was only one sample in the group. The expression pattern of the three marker genes in all tumor samples was shown in supplementary Figure  1 in the Supplementary Material available online at http://dx.doi.org/10.1155/2015/901303.

### 2.2. Copy Number Data Analysis

Level 3 copy number data was downloaded for breast tumor samples from TCGA (platform: Affymetrix SNP6) [6]. As the CNV sizes are quite different across the tumor samples, the CNV profiles were further broken into gene basis. To enable the gene based analysis, the Bioconductor package CNTools was used to map the segmented copy number data of TCGA samples to genes [14], and each gene corresponds to only one CNV segment. The mean copy number profile of each group of the TCGA samples was obtained by calculating the mean signal of each gene across all tumor samples in this group. Copy number data (gene level) for cancer cell lines was obtained from CCLE (platform: Affymetrix SNP6) [7]. As reported by TCGA and CCLE, the significant focal copy number alterations in individual tumor samples/cancer cell lines were identified from segmented data using GISTIC [15].

Four classes of abnormal segments were considered based on their estimated copy number [16]:

1. single copy deletion (copy number < 1.5),
2. double copy deletion (copy number < 0.5),
3. gain of copy number (copy number > 2.5),
4. amplification (copy number > 3.5).

### 2.3. Gene Expression Data Analysis

We used data from the Agilent G4502A_07 platform for TCGA, with measurements of 17,814 genes. Differentially expressed genes were selected based on the fold change of gene expression between each groups of tumor samples and the control (normal group) under the cutoff of |log⁡2foldchange| > 1 [17]. The overexpression/underexpression frequency was calculated for each gene in each tumor group. For example, gene A was overexpressed in ER group as compared to the normal group, and then the proportion of tumor samples in ER group with expression value of gene A higher than the mean expression value of gene A in normal group was defined as the overexpression frequency of gene A in ER group.

CCLE expression data was obtained using Affymetrix U133 Plus 2.0 Arrays, with measurements of 18,926 genes. Differentially expressed genes were selected based on the fold change of gene expression between each cell line and the average of expression value of all the cell lines [17].

For the comparison between gene expression data from TCGA and CCLE, robust *z*-scores (median-centered expression values divided by the median absolute deviation) were derived separately for the two data sets from CCLE and TCGA, and only common genes were remained.

### 2.4. Gene Set Functional Enrichment Analysis

Gene set enrichment analyses were performed for the functional annotation of the differential expressed genes. Functional Annotation Tools in DAVID Bioinformatics Resources [18] were used to carry out these analyses. Those gene ontology biological process terms with *P* value less than 0.05 and genes more than two were considered as significant enriched functions for further analysis.

### 2.5. The Construction of “Pathway of the 384 Genes in Breast Tumors”

First, pathways closely related to breast cancer were collected via NCI website (http://www.cancer.gov/) and literature review, and they are Estrogen Signaling pathway, ERBB pathway, PI3K/Akt/mTOR Signaling pathway, p53 Signaling pathway, Ras Signaling pathway [19], Notch Signaling pathway [20], Wnt Signaling pathway [21], and NFkB pathway [22]. These pathways were retrieved from KEGG pathway database [23] and compiled into a big pathway via the overlapping elements.

### 2.6. Rank Aggregation

Two ranking lists derived from copy number profiles and gene expression profiles were fused into one ranking list using R package RankAggreg [24]. Cross Entropy Monte Carlo (CE) algorithm together with Spearman distance was used to perform the rank fusion. The maximum number of iterations was set as 1000.

## 3. Result

### 3.1. Genomic Characteristics of Breast Tumor

#### 3.1.1. Copy Number Variations in Breast Tumors

**Figure 1:**
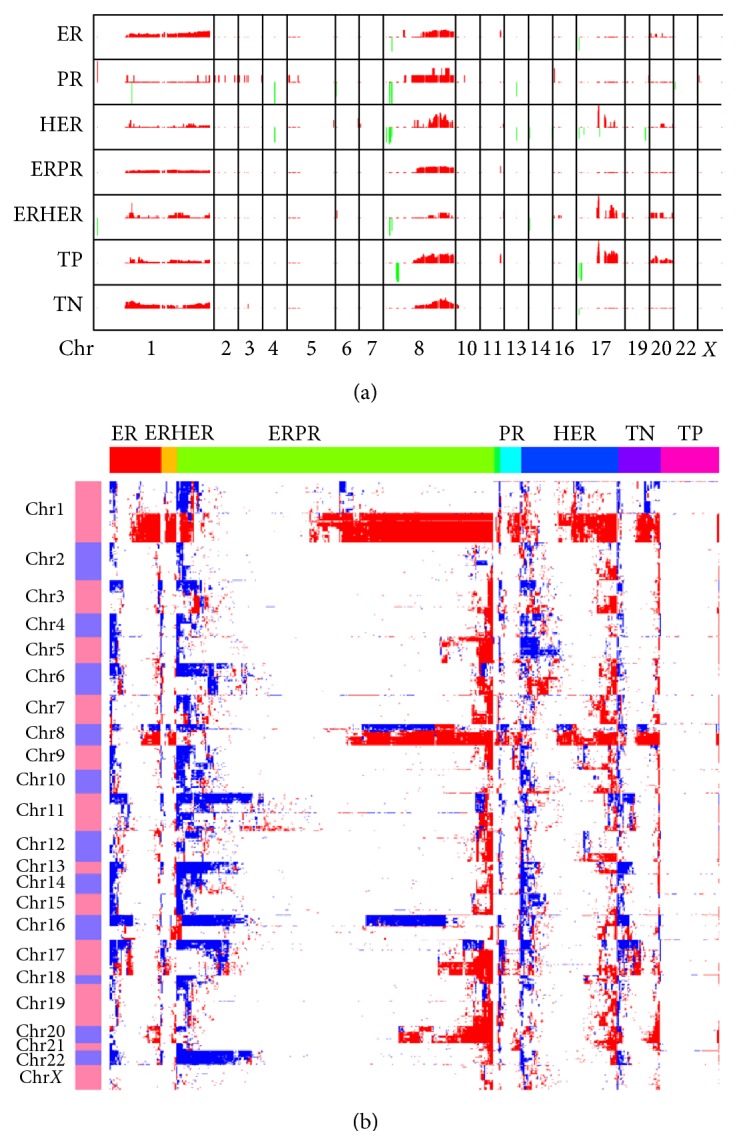
(a) DNA copy number change profiles in each group of breast tumor samples. The CNVs frequency of the whole genome was calculated, the gains of copy number were marked in red, and the losses were marked in green. The *y*-axis in each subgraph represents the frequency of the copy number gain/loss of the corresponding gene. (b) Clustering of the CNV data. The CNVs on each chromosome in each sample group were clustered separately. The gains of copy number were marked in red and the losses were marked in blue.

The TCGA and other groups have made great effort to explore the genomic landscape of breast cancer [6, 25]. After classifying the tumor samples from TCGA, we found that, as compared to the normal samples, the tumor samples in other groups show similar copy number variation (CNV) pattern (supplementary Figure  2). Then, we obtained 2,426 genes with CNVs for all groups (supplementary Table 1). It is noteworthy that, for all the groups, the majority of the genes are undergoing frequent copy number gain (Figure 1). Chromosomes 1, 8, 17, and 20 contained most of the genes with CNVs. According to previous studies [6, 26], many genes on chromosomes 8 and 17 show copy number gain, such as MYC on chromosome 8q24, and HER2 as well TOP2A on chromosome 17q21.1.

#### 3.1.2. Differentially Expressed Genes in Breast Tumors

Totally, there were 4,843 differentially expressed genes (DEGs) for all groups of tumor samples from TCGA (supplementary Table  2). 399 of the DEGs were overexpressed in all the tumor groups, while 588 of them were underexpressed in all groups (supplementary Figures  3 and 4). There were only 5 overexpressed genes and 14 underexpressed genes unique for ER group, while there were 254 overexpressed genes and 219 underexpressed genes unique for TN group (supplementary Figures  3 and 4). Then, the overexpression/underexpression frequency was calculated for each of the 4,843 genes in each group. Notably, 413 of the genes differ greatly in these tumor sample groups (the deviation between the highest and the lowest frequency of the gene across the groups is bigger than 1), and they were significantly enriched in regulation of hormone levels and cell adhesion.

#### 3.1.3. Genes with Correlations between Copy Number and Expression

**Figure 2:**
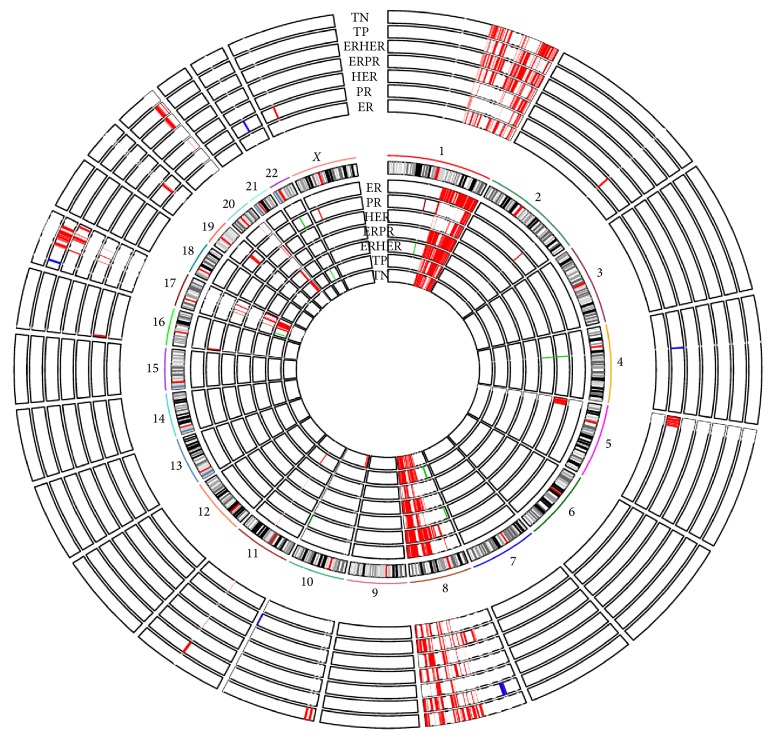
Copy number variation value and expression value of the 384 genes. The 7 circles inside represent the copy number variation of the 384 genes in the 7 tumor groups. The gains of copy number were marked in red and the losses were marked in green. The genes were arranged in chromosomal order (chr1→chr *X*). The circular rings denote different tumor groups (from outside to inside: ER, PR, HER, ERPR, ERHER, TP, and TN). The 7 circles outside represent the expression value of the 384 genes in the 7 tumor groups. The overexpressed genes were in red and the underexpressed ones were in blue. The genes were arranged in chromosomal order (chr1→chr *X*). The circular rings denote different tumor groups (from outside to inside: ER, PR, HER, ERPR, ERHER, TP, and TN).

**Figure 3:**
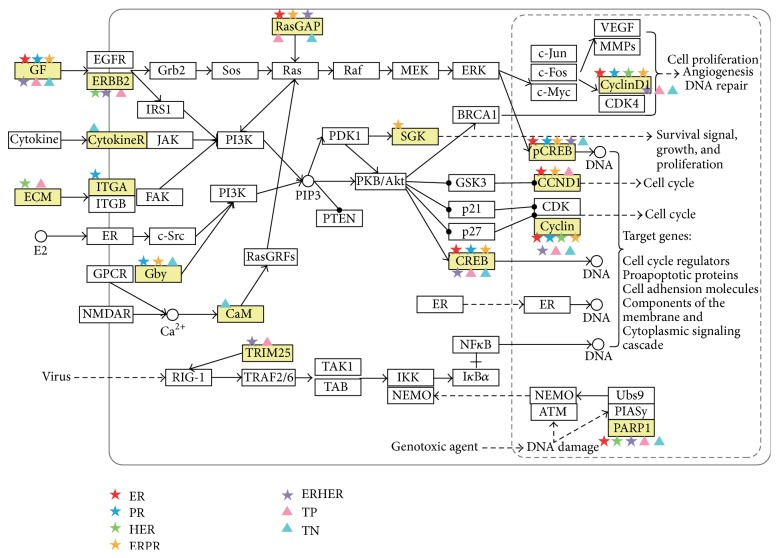
Pathway of the 384 genes in breast tumors. The yellow boxes are the genes that showed copy number change associated with alteration in their expression for all tumor sample groups. The five-pointed stars or triangles with different colors denote the genes in different breast tumor groups.

We found that totally 384 individual genes show copy number change associated with the alteration in their expression for all tumor sample groups (Figure 2 and supplementary Table  3). The majority of these genes were distributed in chromosomes 1, 8, and 17, which is not surprising, as most of the genes with CNVs were concentrated in these chromosomes. The genes with high copy number change also show high gene expression change, such as ERBB2, PSMD3, and TCAP. Altogether these genes are significantly enriched in biological processes related to cell cycle. Amplified (and overexpressed) genes are prime therapeutic targets. For example, the use of the drug trastuzumab against ERBB2 has been shown to improve breast cancer survival rates alone or in combination with other treatments [27–29]. The amplified genes with overexpression in each tumor sample groups might be the potential therapeutic targets for the specific tumor type, such as CCND1, CCNE2 for the ER group and E2F5, EIF2C2 for the PR group. 23 of these genes are distributed in the pathways which are closely related to breast cancer: ERBB pathway, PI3K/Akt Signaling pathway, NFkB pathway, and so forth (Figure 3) whereas whether these genes are druggable needs further exploration.

### 3.2. Correlation between Breast Cancer Cell Lines and Tumor Samples

#### 3.2.1. Characteristics of the Breast Cancer Cell Lines

**Figure 4:**
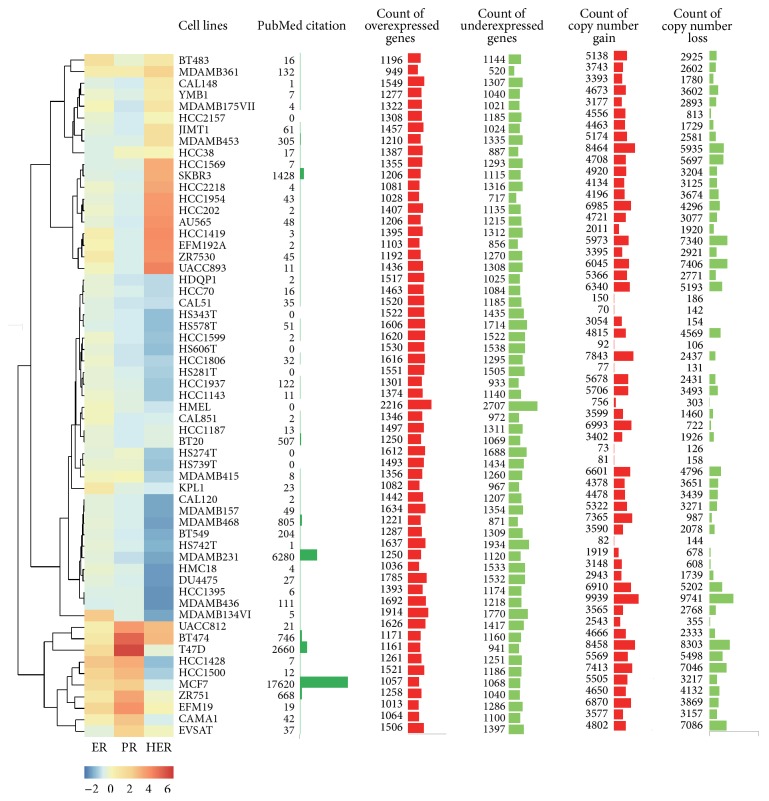
General information of the 59 breast cancer cell lines. The fold change values of ER, HER2, and PR in these cell lines were summarized in a heat map, with blue indicating low fold change value and orange indicating high fold change value.

Among the 59 breast cancer cell lines in CCLE dataset, MCF7, MDA-MB-231, and T-47D are the three most used cell line models for breast cancer account for 82% of current PubMed citations out of the 59 analyzed cell lines (Figure 4). The presence or absence of expression of ER, HER2, and PR in these cell lines was shown in Figure 4, and accordingly, the cell lines were clustered into three parts. These cell lines were also classified into 7 groups as for the breast tumors. The cell lines within the same group show quite different copy number pattern (supplementary Figure  5). The number of overexpressed/underexpressed genes and the count of genes with copy number changes in each cell line were also shown in Figure 4. In general, most of the cell lines have more genes with CNVs rather than DEGs, while CAL51, HS343T, HS606T, HS281T, HMEL, HS274T, HS739T, and HS742T have more DEGs rather than genes with CNVs.

#### 3.2.2. Comparing the Breast Cancer Cell Lines to the Tumor Groups

**Figure 5:**
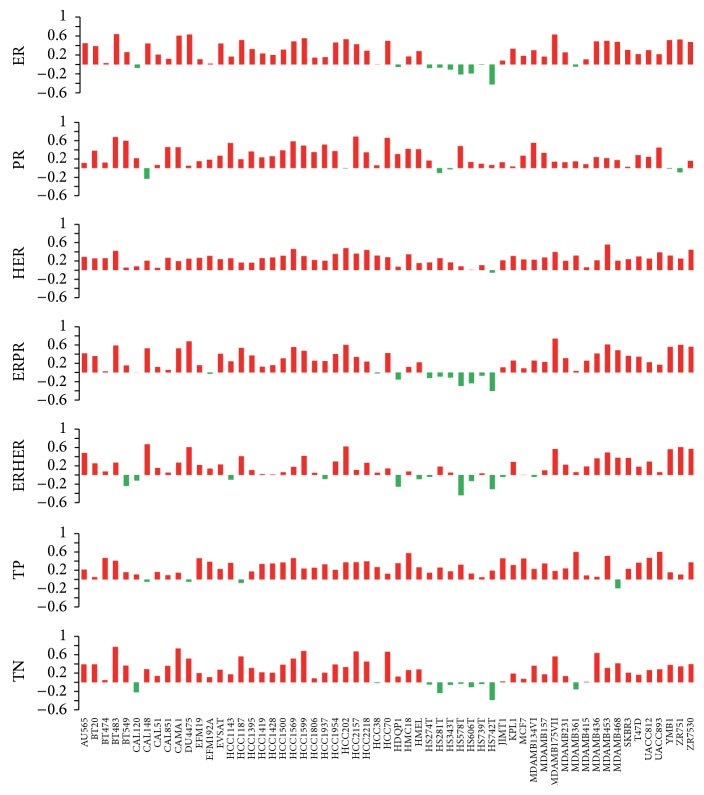
Correlation between the 59 breast cancer cell lines and the tumor sample groups using copy number data.

For each cell line, the copy number profiles were compared with the mean copy number profile of each tumor sample group by calculating Spearman correlation coefficients using the 2,426 genes with CNVs profiles in the tumor samples (Figure 5). In this way, we obtained the correlation between each of the cell lines and the different tumor groups. 12 cell lines (e.g., BT20, BT474, EFM19, etc.) show high correlation with their preclassification indicated by the presence or absence of expression of ER, HER2, and PR in the cell line. We surprisingly found that the most cited three cell lines MCF7, MDA-MB-231, and T-47D do not show high correlation with the preclassified tumor group. Additionally, some cell lines (HS343T, HS606T, HS739T, and HS742T) show low correlation to any one of the tumor groups. This is probably due to the fact that these established breast cancer cell lines are not derived from the primary breast tumors but from tumor metastases. This means that these cell lines are derived from more aggressive metastatic tumors, rather than the primary lesion [1]. Besides, cell lines are purer than tumor samples, which tend to be contaminated with stromal cells [4]. In addition, cell lines are prone to genotypic and phenotypic drift during their continual culture, especially those that have been deposited in cell banks for many years [30].

**Figure 6:**
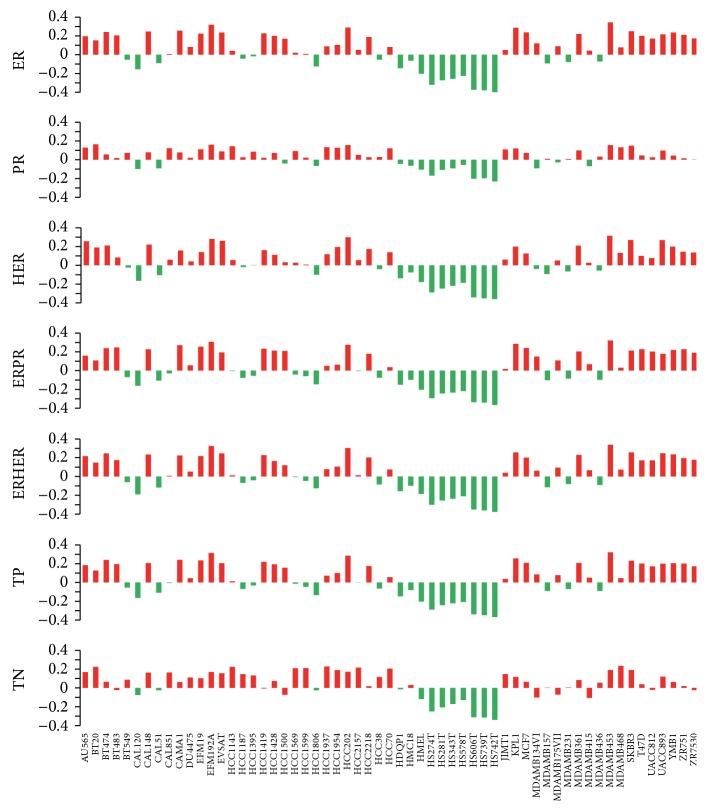
Correlation between the 59 breast cancer cell lines and the tumor sample groups using gene expression profiles.

Then, the gene expression profiles of each cancer cell line were compared with the mean gene expression profile of each tumor sample group by calculating Spearman correlation coefficients using the 4,843 genes differentially expressed in the tumor samples (Figure 6). In this way, we obtained the correlation between each of the cell lines and the different tumor groups. As a whole, the correlation between cell lines and tumor sample groups using gene expression profiles is in accordance with that revealed using CNVs data. The difference is that the correlation values are lower than those calculated using CNVs information but with higher concordance (29 cell lines) with the classification indicated by the presence or absence of expression of ER, HER2, and PR in the cell line. HS343T, HS606T, HS739T, and HS742T also show low correlation to any one of the tumor groups.

**Figure 7:**
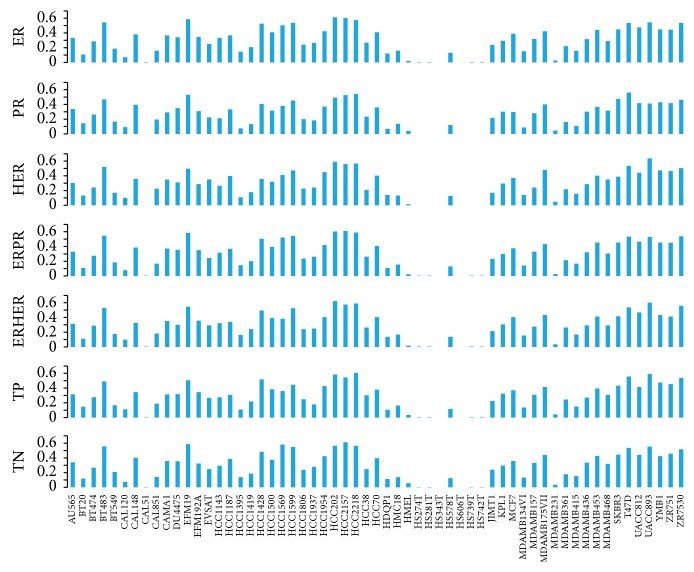
The overlap ratio of genes shows copy number change associated with alteration in their expression between each breast cancer cell lines and the tumor sample groups.

Additionally, we examined the overlap ratio of genes that showed copy number change associated with alteration in their expression between each breast cancer cell line and each tumor sample group (Figure 7). This ratio could also indicate the correlation between cancer cell lines and different tumor samples, as it shows high consistency with that only by copy number profiles or gene expression profiles.

### 3.3. Ranking of the Breast Cancer Cell Lines as Candidate Models for Certain Tumor Group Study

Breast cancer is a complex disease that manifests as a result of coordinated alterations on genomic, epigenomic, and proteomic levels. Therefore, it is important to take into account the multiple datasets together to optimize strength of biological information across multiple assays relevant to breast cancer. With the accumulated copy number profiles and gene expression profiles for different cancer cell lines and tumor samples, we could evaluate whether a certain breast cancer cell line is a good model for a specific tumor group by integration of these two aspects of information. We designed a ranking aggregation model of the cell lines according to their correlation with each tumor group based on the integration of copy number profiles and gene expression profiles. First, the breast cancer cell lines were ranked in descending order of their similarity with each tumor group using copy number profiles and gene expression profiles, respectively. Then, for each tumor group, the two derived ranking lists of the breast cancer cell lines were fused into one ranking list using R package RankAggreg [24]. In this way, the good cell line models for each tumor groups were picked out from the 59 breast cancer cell lines.

For each tumor group, the cell lines ranked in the top resemble the tumor group best and might be the best cell line models for laboratory studies. Suggested by the final ranking list, the best three cell line models for each breast tumor group were listed as follows: CAMA1, BT483, and HCC202 (ER group); HCC70, HCC1143, and HCC1937 (PR group); MDAMB453, HCC2218, and UACC893 (HER group); MDAMB453, CAL148, and ZR751 (ERHER group); HCC202, BT483, and ZR751 (ERPR group); MDAMB453, MDAMB361, and UACC893 (TP group); HCC1599, HCC70, and HCC1569 (TN group). There results may provide useful clues for future personalized breast cancer study.

### 3.4. Comparing All the Cancer Cell Lines with Breast Tumor Samples

Similarly, we also evaluated the correlation between all the cancer cell lines in CCLE and breast tumor sample groups, using the copy number information and gene expression profiles (supplementary Tables  4 and 5). From the perspective of either copy number or gene expression profiles, respectively, some breast cancer cell lines were ranked with high correlation with any of the breast tumor groups while, interestingly, we also identified that some lung cancer cell lines and ovary cancer cell lines also present high correlation with at least one of the breast tumor groups.

## 4. Discussion

### 4.1. Breast Tumor Sample Groups Differ Greatly in the Regulation of Hormone Levels and Cell Adhesion

413 of the DEGs differ greatly in the frequency of overexpression/underexpression across the different tumor sample groups. After conducting the gene set functional enrichment analysis, we found these genes were significantly enriched in biological processes including the regulation of hormone levels and cell adhesion. The enrichment in the regulation of hormone levels is expected. As cell adhesion is related to cancer metastasis, we checked the literatures and found that different breast cancer subtypes show disparity in metastasizing to different sites [31, 32]. However, the classification of breast cancer into subtypes does not typically inform about metastatic behavior. These genes (COL9A1, ITGB8, ITGB6, TTYH1, RET, etc.) enriched in the cell adhesion may serve as important indicators of different types of breast cancer. Due to limited information in this field, the roles of these genes in manipulating the tendency of breast cancer metastasis to different sites need to be further studied.

### 4.2. Correlation between Breast Cancer Cell Lines and the Tumor Samples

The integrative genomic study of copy number profiles and gene expression profiles collected on the same set of cancer cell lines and patient samples could serve as an efficient way to depict the characteristics of different tumor types and cancer cell lines. Besides, the integrated investigation of the two perspectives also provides a guide to reveal the relationship between breast cancer cell lines and the tumor samples, as well as selecting the suitable cell lines for the corresponding breast tumor group. In general, the correlation between the cancer cell lines and the tumor sample groups indicated by the two aspects was consistent with each other. The association between copy number variations and gene expression has been investigated by several research groups [33, 34]. As DNA copy number variations (CNVs) are important influential factors for altered gene expression levels in cancer, the observed high consistency was expected.

Some of the cancer cell lines have high correlation with the preclassified tumor group based on the presence or absence of expression of ER, HER2, and PR in the cell line, while a big part of them does not show this tendency, including the most used MCF7, MDA-MB-231, and T-47D. According to ATCC (http://www.atcc.org/) which is one of the largest biosources in the world and offers investigators a complex array of human, animal, insect, fish, and stem cell lines, these three cell lines are not from primary breast cancer but are metastatic breast cancer cell lines derived from pleural effusion. Some of the cell lines (HS343T, HS606T, HS739T, and HS742T) have low correlation to any one of the primary tumor groups either calculated using copy number profiles or gene expression profiles. The low correlation probably lies in that they are not originated from primary tumors, or maybe these cell lines were contaminated during their continual culture.

Indicated by the fused rank based on the similarity of copy number profiles and gene expression profiles, the most resemble breast cancer cell lines were picked out as the good models for different tumor groups. Further evidences might be identified by investigating mutation profiles, proteomics data, and so forth.

### 4.3. Lung Cancer Cell Lines and Ovary Cancer Cell Lines Show High Correlation with the Breast Tumor Samples

By evaluating the correlation between other cancer cell lines in CCLE with the breast tumor groups, we found some of the lung cancer cell lines and the ovary cancer cell lines show high relevance with the breast tumors. In the systematic analysis of the genomic characteristics of breast tumors, the similarity between ovary tumors and lung tumors was observed [6]. The high correlation between some of the ovary/lung cancer cell lines and the breast tumors was understandable. In addition to the similar CNV profile (e.g., common gains in chromosomes 1, 8, 17, and 20) and gene expression profile (e.g., overexpression of AKT3, MYC) between the breast tumor samples and the ovary/lung cancer cell lines, there are some other commonalities between them. For example, breast tumors and ovary tumors have common risk factors including hormone therapy, obesity, and inherited genetic risk such as BRCA1 and BRCA2 [35, 36]. For breast tumors and lung tumors, they have high frequency of TP53 mutations, EGFR mutation, and so forth [37].

## 5. Conclusion

In this paper, we investigated the correlation between different groups of primary breast tumors and breast cancer cell lines using copy number profiles and gene expression profiles. Although the relevance between tumors and cancer cell lines seems not very high, while considering their ease of use, there is no doubt that established cell lines will continue to be used as models for breast cancer. Our study is expected to provide a useful guide for researchers to understand the limitations of the cells and select the suitable cell lines as the tumor model for better investigation of cancer mechanism.

## Supplementary Material

The gene expression pattern of ER, PR, HER2 and the global copy-number pattern of the breast tumor samples are given in Supplementary figure 1 and Supplementary figure 2. The numbers of over-expressed/underexpressed genes for each tumor groups as well as the overlapped genes of them are shown in Supplementary figure 3 and Supplementary figure 4. The copy-number pattern for the breast cancer cell lines is shown in Supplementary figure 5. The 2,426 genes with CNVs and the 4,843 differ-entially expressed genes (DEGs) for all groups of tumor samples are listed in Supplementary table 1 and Supplementary table 2. The 384 individual genes showed copy number change associated with alteration in their expression for all tumor sample groups are listed in Supplementary table 3. The cor-relation between all the cancer cell lines in CCLE and breast tumor sample groups using copy-number information or gene expression profiles are given in Supplementary table 4 and Supplementary table 5.
